# α-Glucosidase Inhibitory Activity of Polyphenols from the Burs of *Castanea mollissima* Blume

**DOI:** 10.3390/molecules19068373

**Published:** 2014-06-19

**Authors:** Jianwei Zhang, Shan Zhao, Peipei Yin, Linlin Yan, Jin Han, Lingling Shi, Xiaojing Zhou, Yujun Liu, Chao Ma

**Affiliations:** 1 National Engineering Laboratory for Tree Breeding, College of Biological Sciences and Biotechnology, Beijing Forestry University, Beijing 100083, China; 2 Beijing Key Laboratory of Forest Food Processing and Safety, College of Biological Sciences and Biotechnology, Beijing Forestry University, Beijing 100083, China; 3 Institute of Chemistry, Chinese Academy of Sciences, Beijing 100190, China; 4 State Academy of Forestry Administration, Beijing 102600, China

**Keywords:** *Castanea mollissima* Blume, polyphenols, α-glucosidase, diabetic, ellagitannins

## Abstract

Polyphenol extracts from the burs of *Castanea mollissima* Blume (CMPE) exhibited potential antioxidant and hypoglycemic activities. The α-glucosidase inhibitory activities of CMPE were assessed as a means of elucidating the mechanism behind its hypoglycemic activities. *In vitr*o studies showed that CMPE significantly inhibited both yeast α-glucosidase, through a noncompetitive mode with an IC_50_ of 0.33 μg/mL, and rat intestinal α-glucosidase. *In vivo* studies revealed that oral administration of CMPE at doses of 600 mg/kg significantly reduced postprandial blood glucose levels by 27.2% in normal rats following sucrose challenges. Gel permeation chromatography revealed that CMPE exhibited typical characteristics of high-molecular-mass polymers with mean (Mn) and weight (Mw) average molecular weights of 35.4 and 50.7 kDa, respectively, and a polydispersity (Mw/Mn) of 1.432. Acid hydrolysis analysis indicated the presence of ellagitannins. These data suggest that CMPE, enriched with ellagitannins, would be an efficacious dietary supplement for diabetes management through the inhibition of alpha-glucosidase.

## 1. Introduction

Diabetes mellitus, one of the most common metabolic diseases, is caused by a deficiency in insulin levels; type 2 diabetes is characterized by hyperglycemia due to defects in insulin secretion, action, or both [1,2] and has rapidly become a physical and mental burden that lowers the quality of life and results in high rates of mortality and disability. Balancing postprandial blood glucose levels is extremely important, since postprandial hyperglycemia is considered more dangerous than fasting blood glucose [3]. α-Glucosidase can release glucose by hydrolyzing linear and branched isomaltose oligosaccharides, resulting in postprandial hyperglycemia. Therefore, identifying and characterizing the inhibitors of α-glucosidase that can be used therapeutically is important [4,5]. Commercial α-glucosidase inhibitors, such as acarbose and voglibose, have been used to treat diabetes, but they exhibit side effects, including liver disorders, flatulence, abdominal pain, renal tumors, hepatic injury, acute hepatitis, abdominal fullness, and diarrhea [6,7,8,9,10,11]. Recently, a great deal of attention has been paid to natural extracts exhibiting α-glucosidase behaviors, such as those from grape skins, wheat bran and germ, and guava leaves [5,12,13]

Over the past few years, interest in ellagitannins and ellagic acid (EA) has increased due to their properties as micronutrients [14]. Fruits, especially berries and nuts, are rich sources of ellagitannins and EA, which usually provide the characteristic tastes of the fruits and their products [14]. High ellagitannin and EA contents have been reported in strawberries, cranberries, blueberries, blackberries, and chestnuts [15,16]. Ellagitannins and EA have been studied primarily with regard to their positive effects on human health and for their physiological properties such as antitumor, anti-peroxidase, antiviral, antioxidant, and anti-foodborne pathogen and antimutagenic activities [17]. Recently, polyphenol extracts from the burs of *Castanea mollissima* Blume (CMPE), which is native in China and cultivated in North American and Asia as an economic crop, were shown to exhibit antioxidant, hypolipidemic, and hypoglycemic properties [2]. However, many mechanisms exist by which an oral therapeutic agent can exhibit hypoglycemic properties, including insulin sensitization, stimulation of insulin release, and the inhibition of α-glucosidase [18]. In 2010, a 75% ethanol extract of *C. mollissima* was shown to exhibit a potent α-glucosidase inhibitory activity [19]. We presume that CMPE may exert hypoglycemic effects through the same mechanism. The objective of this study was to evaluate the latent capacity of CMPE as an α-glucosidase inhibitor for the *in vitro* and *in vivo* prevention and treatment of diabetes.

## 2. Results and Discussion

### 2.1. Gel Permeation Chromatography of CMPE

**Figure 1:**
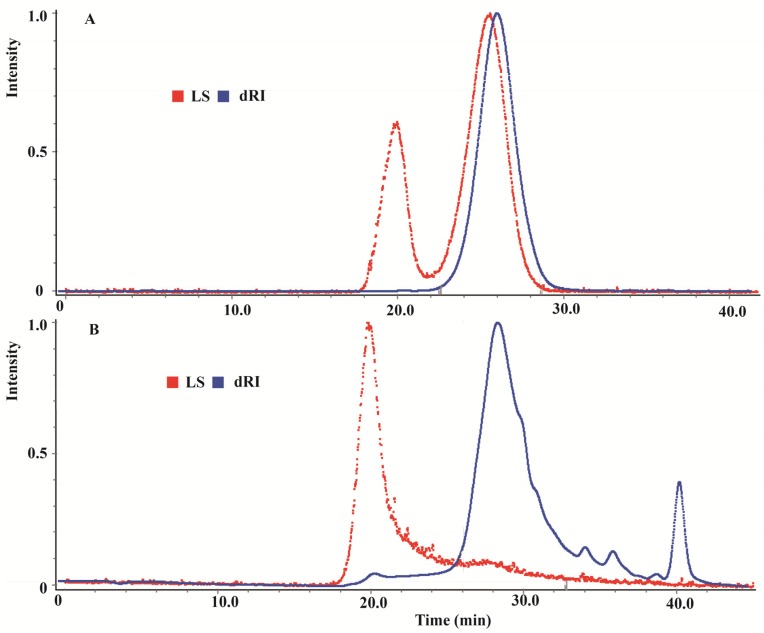
Light scattering (LS) and differential refractometer (dRI) gel permeation chromatogram of (1,3)(1,4)-β-glucan standard (**A**) and CMPE (**B**).

In a previous study, the fraction of extract responsible for the observed antioxidant activities of CMPE contained primarily hydrolyzable tannins [20]. Gel permeation chromatography (GPC) was used to elucidate the detailed structural characteristics of the hydrolyzable tannins in CMPE. Chromatograms of the (1,3)(1,4)-β-glucan standard and CMPE are presented in Figure 1.

**Figure 2:**
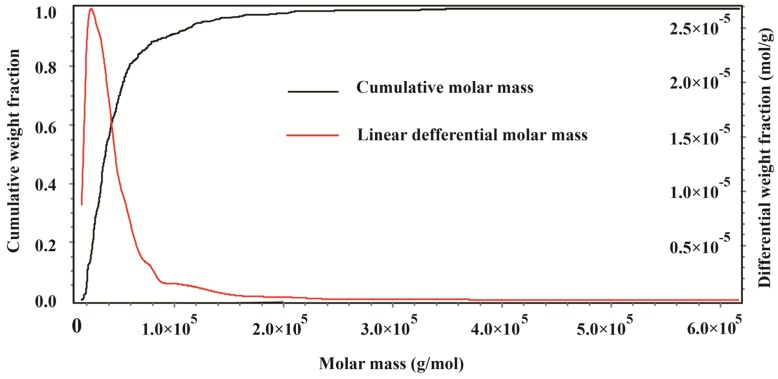
Molecular weight distribution obtained by gel permeation chromatogram for CMPE.

Figure 1A shows that the chromatogram of the (1,3)(1,4)-β-glucan molecular weight standard contained two distinct peaks in the light-scattering GPC (LS-GPC) data and a single peak in the differential refractometer GPC (dRI-GPC) data. The weight average molar mass (Mw) was 30.19 kDa, the number average molar mass (Mn) was 22.80 kDa, and the Z average molar mass (Mz) was 40.49 kDa. The polydispersity index (PDI), as a measure of the distribution of molecular mass in a given polymer sample and calculated as Mw/Mn, was 1.324. Figure 1B shows a single peak in the LS-GPC chromatogram of CMPE, along with a set of major and minor peaks in the dRI-GPC chromatogram. The molecular weight distribution obtained by GPC for CMPE is shown in Figure 2. The mass properties of CMPE were characteristic of typical high-molecular-mass polymers, with an Mw of 50.69 kDa, an Mn of 35.39 kDa, an Mz of 105.8 kDa, and a PDI of 1.432. These data indicate that the major components of CMPE are likely high-molecular-mass polymers. The total tannin and polyphenol contents of CMPE were 622.9 mg and 836.4 mg gallic acid equivalents (GAE) per gram, respectively. Therefore, the major components in CMPE were deduced to be high-molecular-mass polymeric tannins. Previous studies sought to separate the polymeric tannins of CMPE and identify their chemical structures but they were not resolved chromatographically, and only a few reports have successfully clarified the structures of natural oligomeric ellagitannins larger than pentamers [21]. Thus, elucidating the chemical structures of the high-molecular-weight tannins in CMPE, which are much larger than pentamers, is a difficult task.

### 2.2. Acid Hydrolysis Analysis of CMPE

**Figure 3:**
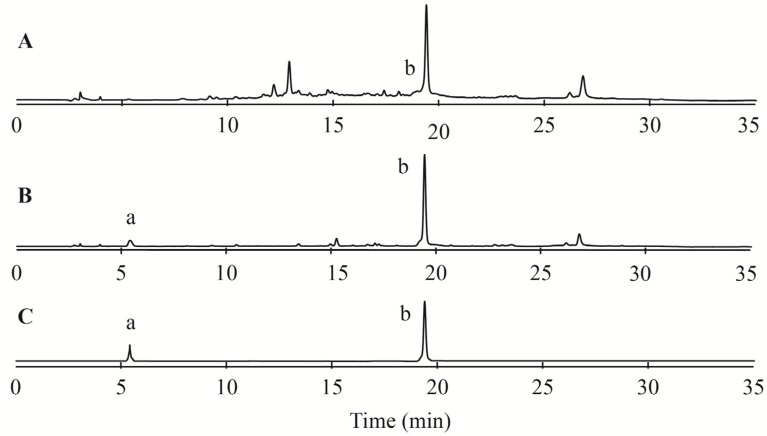
RP-HPLC profiles of CMPE before and after acid hydrolysis. (**A**) CMPE extract before acid hydrolysis; (**B**) CMPE extract after acid hydrolysis; (**C**) gallic acid standard (a) and ellagic acid standard (b).

To quantify the ellagitannin content of CMPE, conventional acid hydrolysis analyses were performed as described by Augilera-Carbo *et al.* [17]. During acid hydrolysis, ellagitannins release hexahydroxydiphenic acid (HHDP), which undergoes spontaneous lactonization to produce EA. Reversed-phase high-performance liquid chromatography (RP-HPLC) chromatograms of CMPE before and after hydrolysis are shown in Figure 3A,B, respectively. Before acid hydrolysis, the EA content of CMPE was about 1.456%, increasing to 16.98% after acid hydrolysis. The gallic acid (GA) content of the extract increased from almost zero before acid hydrolysis to 4.32% after hydrolysis. These data indicated that there were at least 20% ellagitannins in CMPE, accounting for about 1/3 of the tannins contained in CMPE.

### 2.3. Yeast Alpha-Glucosidase Inhibition Assays of CMPE

**Figure 4:**
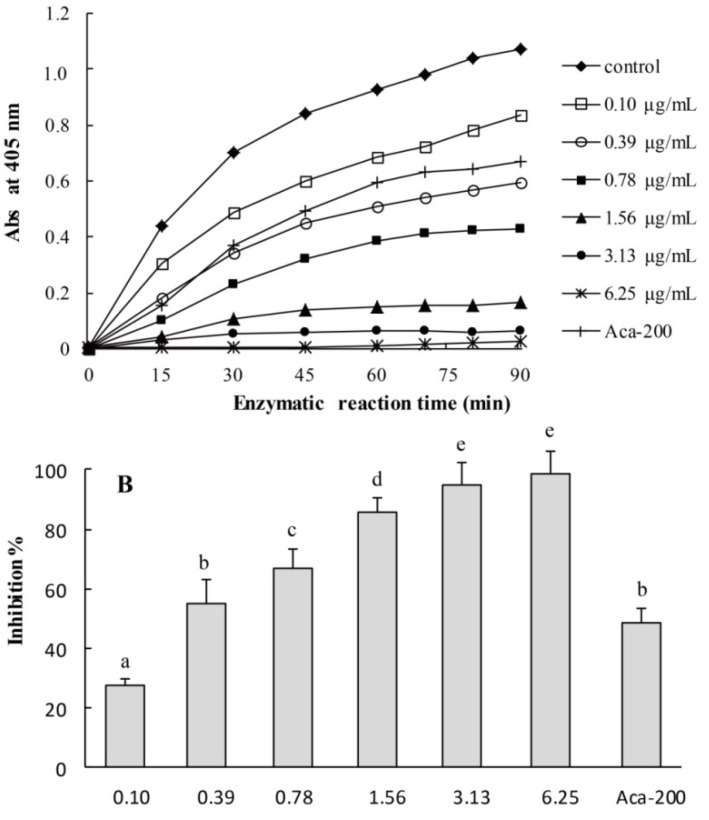
Kinetics of yeast α-glucosidase inhibition by CMPE. (**A**) Kinetics of yeast α-glucosidase inhibition by CMPE at different concentrations. (**B**) Dose-dependent inhibition of CMPE on yeast α-glucosidase. Acarbose (200 μg/mL) is used as positive control and denoted as Aca-200. Bars marked by different letters are significantly different (*n* = 5, *p* < 0.05).

To assess the α-glucosidase inhibition activities of CMPE, yeast α-glucosidase, which is readily available in a pure form and has been widely used in nutraceutical research and medical investigations as a model for screening potential inhibitors [5], was used in the initial screening experiments. Figure 4A shows the time- and dose-dependent inhibition of yeast α-glucosidase as a function of CMPE dose from 0.1 to 6.25 μg/mL over 90 min. CMPE exhibited obvious inhibition on yeast α-glucosidase, which was dependent on both time and dose. By measuring the area under the curve (0–90 min) for each sample and comparing against that of the control in Figure 4A, the α-glucosidase inhibition rate was calculated (Figure 4B). At concentrations as low as 0.1 μg/mL, CMPE showed an obvious inhibition activity. At 3.13 μg/mL, CMPE inhibited more than 90% of the α-glucosidase activity, and at 6.25 μg/mL, the inhibition was nearly complete. The IC_50_ of CMPE was measured as 0.33 μg/mL, which is 613-fold more effective than acarbose (IC_50_ = 200 μg/mL).

**Figure 5:**
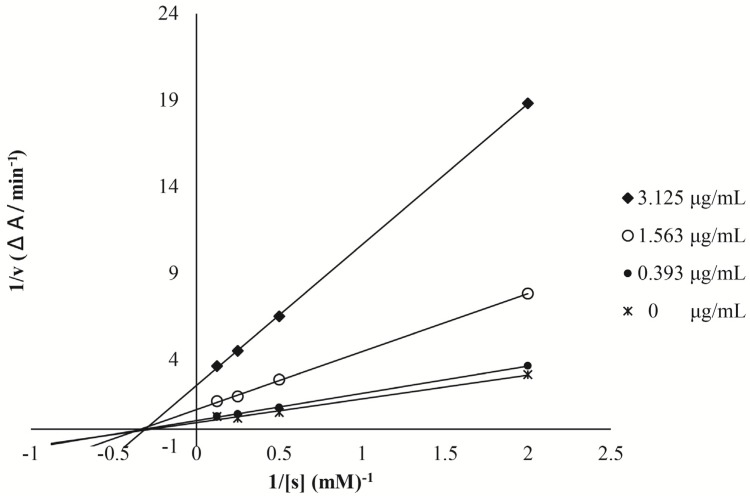
Enzyme kinetic inhibition plots of yeast α-glucosidase inhibition by CMPE. The enzyme reaction was performed with different concentration of *p*NPG (0.5–8 mM) with different concentrations of CMPE.

The mode of inhibition and the inhibition constant (*K_i_*) of CMPE against yeast α-glucosidase were also determined. As shown in Figure 5, Lineweaver–Burke plots show that intersection points corresponding to different doses of CMPE occur on the 1/[s] axis, indicating that increasing the dose of CMPE reduces the maximum rate of the enzymatic reaction without changing the apparent binding affinity of the enzyme for the substrate. The *K_i_* of CMPE was calculated as 1.22 μg/mL.

### 2.4. In Vitro Mammalian α-Glucosidase Inhibition Assays of CMPE

**Figure 6:**
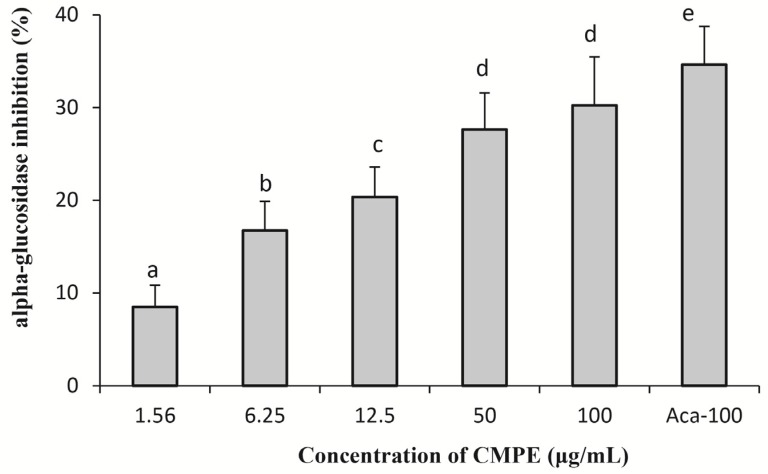
Dose-dependent inhibition of CMPE on rat α-glucosidase. Acarbose (100 μg/mL) is used as positive control and denoted as Aca-100. Bars marked by different letters are significantly different (*n* = 5, *p* < 0.05).

Mammalian intestinal α-glucosidase forms a complex consisting of three individual enzymes: sucrase, maltase, and isomaltase [22]. In the present study, the mammalian intestinal α-glucosidase complex was extracted from the intestines of freshly killed rats. The activity of rat α-glucosidase complex extract was verified using *p*NPG as the substrate and by comparisons with pure yeast α-glucosidase. The mammalian α-glucosidase assay was conducted in a manner similar to that of the yeast α-glucosidase inhibition assay, and the results are shown in Figure 6. The inhibition effects of CMPE against mammalian α-glucosidase were dose-dependent, and CMPE at doses of 1.56, 6.25, 12.5, 50, and 100 μg/mL inhibited the activity of rat α-glucosidase by about 8.5%, 16.8%, 20.3%, 27.7%, and 30.2%, respectively. For comparison, 100 μg/mL of acarbose showed 34.7% inhibition.

### 2.5. In Vivo Mammalian α-Glucosidase CMPE Inhibition Assays

No significant decreases in serum glucose levels were observed in live rats in response to three different doses of CMPE (150, 300, and 600 mg/kg). However, the positive control drug (*i.e*., gliclazide) induced a significant decrease in glucose levels at a dose of 100 mg/kg, which implies that an acute intake of CMPE has no influence on the fasted serum glucose levels in normal rats. 

**Figure 7:**
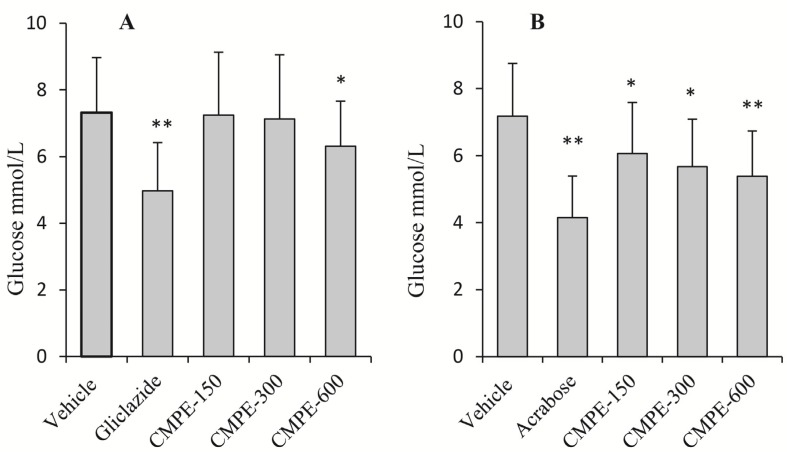
The inhibition of postprandial glycemic response by CMPE in normal rats after glucose (**A**) and sucrose (**B**) challenge (*n* = 5). The fasted rats were orally administrated with vehicle solutions, 100 mg/kg gliclazide, 20 mg/kg acarbose or 150 (CMPE-150), 300 (CMPE-300) or 600 (CMPE-600) mg/kg CMPE. * *p* < 0.05, ** *p* < 0.01 *vs.* vehicle control.

The effects of CMPE and gliclazide on serum glucose levels in glucose-loaded rats are shown in Figure 7A. Oral administration of CMPE at high doses (600 mg/kg) and gliclazide significantly reduced blood sugar 1 h after the glucose challenge. However, no significant changes in blood sugar levels were observed at doses of 150 and 300 mg/kg. These data indicate that at high doses, CMPE depresses postprandial serum levels via a mechanism linked to insulin release, similar to the mechanism by which gliclazide acts. The effects of CMPE and acarbose on serum glucose levels in sucrose-loaded rats are shown in Figure 7B. Decreased serum glucose levels in rats treated with CMPE were dose-dependent with a maximum decline of 27.2% in serum glucose levels relative to the vehicle control. For comparison, acarbose also significantly reduced serum glucose levels. The above data indicate that CMPE improved sucrose tolerance through a mechanism that may be related to its α-glucosidase inhibition activity.

### 2.6. Discussion

As a traditional economically important nut, *C. mollissima* Blume, known as Chinese chestnut, has been the focus of nutritional studies that detail its beneficial roles in human health. Recently, attention has spread from the nut kernel to the shells [23], flowers [24], testas [19], and burs [2,20]. Abundant polyphenols with distinct antioxidant activities have been reported in chestnut burs [25]. To isolate high-purity polyphenols from such burs, macroporous resins were used to separate and enrich the polyphenols into a CMPE. After enrichment, the polyphenol and tannin content of the CMPE reached 836.4 mg and 622.9 mg GAE/g, respectively. GPC analyses revealed that the CMPE exhibited high-molecular-mass polymeric characteristics with a Mw of 50.69 kDa and a PDI of 1.432. Acid hydrolysis of CMPE yielded abundant GA and EA, indicating that a considerable quantity of hydrolyzable ellagitannins exist in CMPE.

In *in vitro* yeast α-glucosidase inhibition assays, CMPE was much more effective (IC_50_ = 0.33 μg/mL) than acarbose (IC_50_ = 200 μg/mL). Enzyme kinetic inhibition plots suggested that CMPE is a non-competitive inhibitor of α-glucosidase. CMPE also significantly inhibits the activity of rat α-glucosidase in a dose-dependent manner. However, the CMPE was much more effective against yeast α-glucosidase than rat intestinal α-glucosidase, which may have been the result of non-covalent bond formation between the various components of CPME and intestinal proteins. The potent *in vitro* α-glucosidase inhibitory activity of CMPE led to further studies exploring whether CMPE could limit or delay the digestion and/or absorption of oligosaccharides, thereby reducing the postprandial glycemic response. Animal studies showed that fasted serum glucose levels were not affected in normal rats after a single-dose administration of CMPE. In a sucrose-challenge experiment, CMPE at all doses resulted in notably reduced serum glucose levels compared to vehicle controls. Thus, the *in vivo* and *in vitro* inhibition activities of CMPE are in agreement.

## 3. Experimental

### 3.1. Plant Materials and Chemicals

Burs of *C. mollissima* were harvested from a chestnut plantation in Qianxi, Hebei Province, China, at the beginning of the harvest season of 2011, and authenticated by Yujun Liu. A specimen with a voucher number (CMB-2011-10-04) was deposited in the herbarium of Beijing Forestry University. The burs were air-dried until reaching equilibrium with the ambient humidity, ground, and transferred to dark surroundings until further use. Yeast α-glucosidase (type I) and *p*-nitrophenyl-α-d-glucopyranoside (*p*NPG) were purchased from Sigma-Aldrich (St. Louis, MO, USA). (1,3)(1,4)-β-Glucan molecular weight standards were obtained from Megazyme International Ltd. (Bray, Ireland). Other chemicals were purchased from Alfa Aesar, Inc. (Ward Hill, MA, USA), unless otherwise specified.

### 3.2. Animals

Special pathogen-free SD rats were purchased from the Laboratory Animal Center of the Academy of Military Medical Sciences, China, and housed in a room under controlled conditions with temperature maintained at 22 ± 3 °C, 30%–60% relative humidity, and a 12-h light–dark cycle during experiments. The animals were fed pelleted food, and tap water was available *ad libitum*. Animals were quarantined and allowed to acclimate for one week prior to experimentation. Throughout the experiments, animals were monitored and maintained in accordance with the Beijing Forestry University ethical recommendations and guidelines for the care of laboratory animals.

### 3.3. Preparation of Polyphenol Extracts of C. mollissima

One kilogram of air-dried *C. mollissima* burs was extracted using 50% aqueous ethanol (2 L × 3 times) in a shaking, constant-temperature water bath at 80 °C. Each extraction was performed for 1 h. The resulting slurries were centrifuged at 5000 *g* for 10 min (model GL 10MD; Xiang-yi, Changsha, China) and the supernatant was collected and combined. The combined supernatant was evaporated until 20% of the original volume remained. The concentrated supernatant was diluted with distilled water to the initial volume. After this replenishment, the supernatant was centrifuged at 5,000 g for 10 min. The supernatant was then separated through a chromatography column (800 mm × 60 mm) packed with HPD 100 macroporous resin. The column was eluted with 2000 mL distilled water, followed by washing with 2,000 mL of 50% (v/v) ethanol/water. Finally, the 50% ethanol eluate was evaporated and lyophilized to derive the CMPE. The total tannin and polyphenol contents of the CMPE were 622.9 mg and 836.4 mg GAE/g, respectively, as determined in accordance with the method described in a previous study [20].

### 3.4. Gel Permeation Chromatography of CMPE

To obtain the molecular weight distribution and average molecular weights, CMPE (5 mg) was dissolved in sufficient water, filtered through a 0.45-μm filter, and analyzed by GPC with a chromatographic system (Waters, Milford, MA, USA) equipped with a 515 HPLC pump, tandem detectors (DAWN^®^ HELEOS™ II 18-angle light-scattering detector; Wyatt Technology, Goleta, CA, USA), and an Optilab^®^ rEX differential refractometer (Wyatt Technology). The columns were SHODEX^®^ SB-804HQ and SHODEX^®^ SB802HQGFC (aqueous GPC) columns (8 × 300 mm for each one, 600 mm total length; Showa Denko K.K., Tokyo, Japan) that were run in tandem. The chromatographic system was maintained at 40 °C and run at a flow rate of 0.5 mL/min with an eluent of 100 mM NaNO_3_ containing 5 mM NaN_3_. The calibration curve was obtained with (1,3)(1,4)-β-glucan molecular weight standards. Values were calculated using OmniSEC 4.6 software (Viscotek, Houston, TX, USA).

### 3.5. Acid Hydrolysis of CMPE

A method described by Pinto *et al.* [26] was applied with slight modifications as a reference for conventional ellagitannin hydrolysis and to analyze the total EA content of CMPE. Briefly, CMPE (50 mg) was suspended in aqueous hydrochloric acid (25 mL, 10%, v/v) and then heated under reflux at 80 °C for 12 h. The hydrolyzed sample was then evaporated to dryness with a Heidolph Instruments (Schwabach, Germany) rotary evaporator at 60 °C. Subsequently, the dried residue was dissolved in dimethyl sulfoxide (DMSO, 0.5 mL) and then filled up to a volume of 500 mL with methanol. Prior to injection into the HPLC system, samples were filtered through 0.22-μm syringe filters (Exapure™; ALYS Technologies, Beijing, China).

Chromatographic analyses of EA were performed on a high-performance liquid chromatograph (LC-20AT; Shimadzu, Tokyo, Japan). The apparatus was equipped with an autosampler (SIL-20A), a UV/VIS detector (SPD-20A), a communications bus module (CBM-20A), two liquid chromatographs (LC-20AT), and a column oven (CTO-10AS). A Diamonsil RP-C18 column (5 μm, 250 × 4.6 mm; Dikma, Beijing, China) was employed for acid hydrolysis analyses of CMPE. A gradient elution program with a mobile phase of 0.2% phosphate buffer (A) and acetonitrile (B) was performed as follows: 0–30 min, A 0.92→0.60 mL/min, B 0.08→0.40 mL/min; 30–40 min, A 0.60 mL/min, B 0.40 mL/min. The acid hydrolysis products were identified by comparisons of their retention times and UV spectra with those of standard solutions of EA and GA (0.1 mg/mL). UV absorbance at 260 nm was used to quantify EA and GA with a 10-μL injection volume. Results were expressed as milligrams (EA or GA) per gram of sample on a dry weight basis.

### 3.6. In Vitro Yeast α-Glucosidase Inhibition Assays

Yeast α-glucosidase enzyme inhibition assays were carried out on 96-well microplates in accordance with the method described by Si *et al.* [27] using *p*NPG as a substrate. Briefly, α-glucosidase (10 μL, 1.0 unit/mL) was mixed with different concentrations of CMPE (100 μL, 12.5, 6.25, 3.125, 1.56, 0.78, 0.39, and 0.2 μg/mL) in a 96-well plate for 10 min at 37 °C. The same volume of 0.1 mM phosphate buffer (pH 6.8) was used as a negative control, and 200 μg/mL acarbose was used as a positive control. To avoid the interference of CMPE, the same volume of 0.1 mM phosphate buffer with a same concentration of CMPE was used as blank control. After incubation for 5 min, 2 mM *p*NPG solution in 0.1 mM phosphate buffer (pH 6.8) (30 μL) was added to quickly initiate the enzyme reaction. The release of *p*-nitrophenol from *p*NPG was monitored by measuring the solution absorbance at 405 nm every 15 min for 90 min with a microplate reader (Bio-Rad, Hercules, CA, USA). The enzyme inhibitory activity was determined by calculating the area under the curve (0–90 min) for each sample and comparing this value with that of the negative control. The logit method was used to calculate the CMPE concentration that would exhibit a 50% inhibition of enzyme activity (IC_50_).

To clarify the inhibition mode of CMPE on yeast α-glucosidase, the enzyme reaction was monitored in the presence of various doses of CMPE (0, 3.125, 1.563, and 0.393 μg/mL) incubated with 1.0 unit/mL α-glucosidase (10 μL), and various doses of *p*NPG (50 μL, 1, 2, 4 and 8 mM). Solution absorbance was measured at 405 nm after a 75-min incubation period. Lineweaver-Burke plots were prepared to display the inhibition pattern. *Ki* and IC_50_ values for CMPE were also determined for acarbose as a positive control.

### 3.7. In Vitro Mammalian α-Glucosidase Inhibition Assays

Rats were fasted for over 24 h with free access to water prior to experimentation. In accordance with a method reported by Zhang *et al.* [5], the intestines of experimental rats were removed and put on ice after the rats had been anesthetized with ether. The α-glucosidase complex was then extracted from the mucous membrane of the small intestine from the duodenum to the jejunum and prepared in ice-cold 0.1 M potassium phosphate buffer (pH 7.0) containing 5 mM ethylenediaminetetraacetic acid (EDTA). After centrifugation, the supernatant was collected as an α-glucosidase extract. Then, the rat α-glucosidase extract (20 μL) was mixed with 0.1 mM phosphate buffer (pH 6.8, 150 μL) and CMPE (50 μL) at concentrations of 50, 6.25, or 1.56 μg/mL. The solutions were incubated at 37 °C for 10 min. After incubation, *p*NPG solution (30 μL, 2 mM) was added to quickly initiate the enzyme reaction, and a microplate reader was used to monitor the absorbance at 405 nm every 15 min until the end of the reaction. The same volume of 0.1 mM phosphate buffer (pH 6.8) was used as a negative control, and 100 μg/mL acarbose was used as a positive control.

### 3.8. Influence of CMPE on in Vivo Serum Glucose Levels

To determine the effects of CMPE on fasted serum glucose levels in rats, all rats (six per group) were fasted for 12 h before receiving 150, 300, or 600 mg/kg of CMPE by gastric intubation. Rats in the vehicle control group were given 0.5% sodium carboxymethyl cellulose (CMC-Na). Gliclazide (100 mg/kg) was used as the positive control drug. Serum glucose levels were monitored at 0, 1, and 2 h after drug administration by tail bleeding and by the glucose oxidase method.

Also, to determine the effects of CMPE on postprandial serum glucose levels in glucose- or sucrose-loaded rats, all rats (six per group) were fasted for 12 h before receiving 150, 300, or 600 mg/kg CMPE by gastric intubation. Rats in the vehicle control group were given 0.5% CMC-Na. In glucose-loaded rats, gliclazide (100 mg/kg) was used as the positive control drug, and in sucrose-loaded rats, acarbose (20 mg/kg) was used as the positive control drug. One hour after administration, 2 g/kg glucose or sucrose was given by oral administration. Serum glucose levels were determined 1 h after glucose loading.

### 3.9. Statistical Analyses

All results expressed herein are the mean ± standard deviation (S.D.). Significant differences between groups were identified using the Student’s *t*-test. A *p*-value of less than 0.05 was considered to be significant.

## 4. Conclusions

CMPE, containing abundant ellagitannins, inhibited yeast and rat α-glucosidase *in vitro* and exhibited postprandial blood glucose suppressing effects *in vivo*. The results therefore demonstrate the potential of CMPE as a novel dietary phytonutrient for diabetes management with inhibition activities similar to those of green tea [28] or grape skin extracts [5].
